# Development and validation of the inclusive sport attitudes scale for pre-service sport professionals (ISA-PS)

**DOI:** 10.3389/fspor.2026.1917186

**Published:** 2026-07-29

**Authors:** Zsuzsanna Anti, Katalin Kälbli, Ilona Bodnár, İsmail İlbak, Lilla Lendvai, Judit Gombás, Mónika Szigethy, Júlia Patakiné Bősze, Szilvia Boros, Gusztáv József Tornóczky

**Affiliations:** 1 Doctoral School of Education, Faculty of Education and Psychology, ELTE Eötvös Loránd University, Budapest, Hungary; 2 Institute for the Methodology of Special Needs Education and Rehabilitation, Bárczi Gusztáv Faculty of Special Needs Education, ELTE Eötvös Loránd University, Budapest, Hungary; 3 Independent Researcher, Budapest, Hungary; 4 Faculty of Sports Sciences, İnönü University, Malatya, Türkiye; 5 Institute of Intercultural Psychology and Education, Faculty of Education and Psychology, ELTE Eötvös Loránd University, Budapest, Hungary; 6 Institute of Sport Sciences, Faculty of Education and Psychology, ELTE Eötvös Loránd University, Szombathely, Hungary; 7 Institute of Lifestyle and Physical Culture, Faculty of Economics, Health Sciences and Social Studies, Károli Gáspár University of the Reformed Church in Hungary, Budapest, Hungary; 8 Department of Psychology and Health Management, Faculty of Health and Sport Sciences, Széchenyi István University, Győr, Hungary; 9 Physical Education and Exercise Centre, Medical School, University of Pécs, Pécs, Hungary; 10 Institute of Health Promotion and Sport Sciences, Faculty of Education and Psychology, ELTE Eötvös Loránd University, Budapest, Hungary

**Keywords:** attitudes, disability, inclusion, inclusive sport, ISA-PS, pre-service sport professionals, scale validation

## Abstract

**Background:**

Inclusion in sport extends beyond school-based physical education and requires the active engagement of professionals across diverse sport-related fields. Although several instruments have been developed to assess attitudes toward inclusion among physical education teachers and teacher candidates, there remains a lack of psychometrically validated measures designed for the broader population of future sport professionals. This study aimed to develop and validate the Inclusive Sport Attitudes Scale for Pre-Service Sport Professionals (ISA-PS), a theoretically grounded instrument intended for use across sport education pathways and professional contexts.

**Methods:**

The instrument was developed through a multi-stage process involving a systematic review of the literature, expert evaluation, and a qualitative preliminary study with practicing sport professionals. Psychometric evaluation was conducted using data collected from two independent samples of Hungarian university students preparing for diverse sport-related professions (Study 1: *n* = 312; Study 2: *n* = 167).

**Results:**

Principal component analysis identified a four-factor, 16-item structure accounting for 65.2% of the total variance, encompassing positive willingness, negative components for sport professionals, positive components for participants, and barriers to inclusion. Confirmatory factor analysis indicated that a bifactor model provided the best representation of the data (CFI = 0.948, TLI = 0.930, RMSEA = 0.047, SRMR = 0.061), supporting the interpretation of both a general inclusive sport attitude factor and meaningful specific dimensions. Reliability analyses demonstrated good internal consistency for the total scale (Cronbach's *α* = .87; McDonald's *ω* = .94). Consistent with factors known to support successful inclusion, students with ongoing participation in inclusion-related coursework and those reporting repeated interactions with persons with disabilities during coursework demonstrated significantly higher attitude scores.

**Conclusion:**

Together, these findings suggest that the ISA-PS broadens the assessment of inclusive sport attitudes beyond teacher education and school settings. As such, the ISA-PS represents a valid and reliable instrument for assessing inclusive sport attitudes among pre-service sport professionals from different sport-related educational programs.

## 1 Introduction

In contemporary educational and social discourse, the concept and approach of inclusion are not limited to the effectiveness of educating children with special educational needs (SEN) (1, 2). Inclusion also plays a key role in supporting the full participation of persons with disabilities and in ensuring equal access across various domains, including sport (3–5). For persons with disabilities, regular participation in physical activity plays an important role from childhood onward, as its physical, psychological, and social benefits contribute to reducing the physical and societal barriers associated with disability (6). Participation in physical activity and the development of a sport identity have been positively associated with perceived quality of life (7), as well as with self-confidence, self-esteem, and perceived competence (8–10). Beyond these individual benefits, previous research has also demonstrated that shared physical activity involving persons with and without disabilities may contribute to reducing the stigmatization of persons with disabilities and to eliciting more positive emotional experiences among both groups (11–15). In this context, the role of professionals responsible for implementing inclusive practices becomes particularly important. Just as teachers play a crucial role in public education (16–18), sport professionals also bear fundamental responsibility for the successful implementation of inclusion in sport settings (19–21).

Research on inclusive education has highlighted a range of teacher-related factors associated with the successful implementation of inclusion. Frequently examined factors include professional knowledge regarding inclusion (22, 23), practical experience gained through contact with individuals with disabilities (24–26), and attitudes toward inclusion (27, 28). Of these, attitudes have received particular attention in the literature, as educators with positive attitudes facilitate better academic performance for their students, foster more positive self-esteem in learners concerned, and contribute to more favorable perceptions of students with SEN or disabilities among their typically developing peers (27, 29, 30).

Across Europe, numerous universities have incorporated courses focusing on inclusion into education and training programs (31), and in the field of sport these courses are not limited to physical education teachers but also involve coaches, recreation professionals, and sport managers (32). This is particularly important, as these professionals ultimately determine whether persons with disabilities are included in sport activities, and their reluctance may therefore pose a substantial barrier to equal access to sport (4, 33). Despite this, research on inclusive sport continues to focus predominantly on the attitudes of in-service or pre-service physical education (PE) teachers within public education settings, even though examining other future sport professionals would be essential for monitoring the effectiveness and impact of newly integrated training areas as well.

To assess future sport professionals' attitudes toward inclusive sport, a scale was required that met the following criteria: it had to measure attitudes reliably and validly while remaining quick and easy to complete in line with the characteristics of the current student generation. The use of contemporary perspectives and terminology was also an important consideration (e.g., avoiding the term handicapped). As Kielblock and Woodcock (34) demonstrated, scales employing outdated language cannot be rendered applicable through mere word substitution, given the differing contexts in which they were developed. In addition, the multidimensional nature of attitudes was considered a key design principle, allowing for the simultaneous assessment of cognitive, affective, and behavioral components (35), as they differ in their degree of stability (36). A key requirement for the scale was a strong theoretical foundation to ensure that attitudes toward inclusive sport and the corresponding behavioral intentions could be meaningfully related to one another. Consequently, instead of instruments measuring general attitudes toward disability, a scale was required that specifically assesses attitudes toward inclusive activities. Sport represents a distinct context (37) in which professionals often place considerable emphasis on physical characteristics that may be influenced by certain disabilities (38, 39); therefore, the measurement instrument needed to explicitly reflect the sport setting. In addition, to enable the examination of attitudes among sport professionals beyond the field of physical education teaching, the instrument needed to move beyond classroom-based physical education settings and teacher–student relationships, as inclusive sport for persons with disabilities encompasses a broader range of contexts. Furthermore, in consideration of the study sample, a scale applicable to university students was required, avoiding items that can only be evaluated by practicing professionals with formal teaching experience. According to the Theory of Planned Behavior (TPB) (40), a widely applied framework for examining the relationship between attitudes and behavioral intentions, accurate prediction of intention requires that the behavior toward which the attitude is directed be defined as precisely as possible (41). Thus, the scale was required to assess attitudes toward inclusive sport within a clearly defined behavioral context and target population.

During our systematic literature review, we identified nearly one hundred attitude scales; however, for a substantial proportion of these instruments, the criteria of reliability and validity could not be adequately evaluated due to the lack of published psychometric properties. Scales employing outdated terminology were also excluded: *Opinions Relative to Integration of Students with Disabilities* (ORI) (42); *Physical Educators' Attitude Toward Teaching Individuals with Disabilities* (PEATID-III) (43), as they are no longer applicable in contemporary contexts. While several widely used instruments lack explicit theoretical grounding—such as the *Sentiments, Attitudes and Concerns about Inclusive Education–Revised* (SACIE-R) (44) and *the Teachers' Attitudes towards Inclusive Education Scale* (TAIS) (45)—other instruments assess multiple attitude components, including the *Multidimensional Attitudes Toward Inclusive Education Scale* (MATIES) (46) and the *Inclusion Attitude Scale for High School Teachers* (ISHST) (47). In addition, some scales explicitly adopt the TPB as their theoretical framework, such as the *Attitudes Towards Inclusion of Students with Physical Disabilities in Physical Education–Revised* (ATIPDPE-R) (48), MATIES (46), and the *Physical Educators' Judgements about Inclusion* (PEJI) (49). Despite their theoretical foundations, these instruments predominantly include items tailored to in-service professionals and are restricted to public education contexts. Following the recommendations of Fishbein and Ajzen (41), instruments measuring general attitudes toward disability such as *Attitudes Toward Disabled Persons* (ATDP) (50); *Multidimensional Attitudes Scale toward Persons with Disabilities* (MAS) (51), rather than attitudes toward inclusive activities, were also deemed unsuitable.

With specific regard to the sport context, existing research has primarily focused on the attitudes of physical education teachers: ATIPDPE-R (48); *Concerns about Inclusive Education Scale—Physical Education* (CIES-PE) (52); PEJI (49). Although measurement instruments applicable to pre-service teachers are available, these typically include items that cannot be meaningfully interpreted by students enrolled in coaching or recreation-related programs. Among the identified instruments, scales developed specifically to assess coaches' attitudes were likewise excluded, as they either relied on outdated terminology or lacked sufficient published evidence regarding their reliability and validity: *Coaches Attitude Toward Integration Questionnaire* (CATIQ) (53); *Aquatic Instructors' Beliefs Toward Inclusion* (AIBTI) (38).

Existing multidimensional instruments provide a valuable foundation for assessing inclusion-related attitudes. However, our review of existing scales did not identify any instrument in either the national or the international literature that met all the criteria established for assessing future sport professionals' attitudes toward inclusive sport practices. Accordingly, the aim of this study was to develop and psychometrically validate the Inclusive Sport Attitudes Scale for Pre-Service Sport Professionals (ISA-PS), a multidimensional, and theoretically grounded instrument designed to assess attitudes across diverse sport-related educational programs. In addition to establishing its psychometric properties, the applicability of the instrument was explored by examining its associations with selected educational experiences and background characteristics considered relevant to inclusion-related attitudes, including participation in inclusion-related coursework, contact with individuals with disabilities during coursework, and the presence of practical and attitude-shaping course elements.

## 2 Materials and methods

### 2.1 Instrument development of ISA-PS

The development of the Inclusive Sport Attitudes Scale for Pre-Service Sport Professionals (ISA-PS) followed a multi-step procedure. As a first step, a systematic review of the literature was conducted to identify existing instruments assessing attitudes toward disability and inclusion. The review was performed in the EBSCO and Web of Science databases using predefined search terms related to inclusion, disability, attitudes, measurement scales, sport, physical education, and physical activity. The search yielded 1,220 records, of which 133 studies met the inclusion criteria and were retained for analysis. Across these studies, 92 relevant measurement instruments were identified. Of these, 27 scales were selected based on their conceptual relevance to inclusive sport and physical activity contexts. Several recurring content domains were identified across the reviewed instruments, including views on inclusion, perceived impact on participants, perceived abilities and limitations, contextual resources, and professional preparedness.

An initial pool of 214 items was generated from the selected instruments. This pool was subsequently reduced by removing redundant items, items specific to non-sport educational contexts, linguistically outdated or inappropriate formulations, and by merging conceptually overlapping statements. Drawing on the resulting item pool as a conceptual reference, new items were generated in line with the aims of the present study, focusing on attitudes toward inclusive sport practices among pre-service sport professionals. A preliminary set of 45 items was then evaluated by a multidisciplinary expert panel (*n* = 6) with expertise in adapted physical education, special education, sport sociology, intercultural psychology, recreation, and educational sciences. Experts assessed item relevance and clarity and provided qualitative feedback. Based primarily on expert ratings and qualitative consensus, 27 items were retained, with consideration also given to the balanced representation of the three attitudinal components (cognitive, affective, and behavioral) and the five most frequently identified content domains.

To ensure cultural relevance and theoretical alignment with the Theory of Planned Behavior, qualitative data were collected from in-service sport professionals (*n* = 24) using open-ended questions exploring their experiences and perceptions of inclusive sport. Responses were analyzed using content analysis, which revealed three additional context-specific themes reflecting practical constraints, role-related considerations, and perceived professional responsibility. These themes were incorporated into the item pool, resulting in a 30-item attitude scale. In line with Ajzen's recommendations for the development of TPB-based measures (54), six additional items representing subjective norms, perceived behavioral control, and intention toward inclusive sport were developed based on the findings of the qualitative phase. Given the sensitive nature of the topic and respondents' limited prior familiarity (55), all items were rated on a 6-point Likert scale to reduce the tendency to select a neutral midpoint response (56). Prior to completion, participants received standardized instructions and definitions of inclusion and inclusive sport sessions.

Based on the results of the principal component analysis (PCA), a four-factor model comprising 16 items was selected. Although the two-, three-, and four-factor solutions all demonstrated good psychometric properties, theoretical considerations favored the four-factor model as the most appropriate for the aims of the scale development, because it provided the broadest coverage of the identified content domains while preserving balanced representation of the cognitive, affective, and behavioral components within the retained item set. The four factors were labeled Positive Willingness (5 items), Negative Components for Sport Professionals (5 items), Positive Components for Participants (3 items), and Barriers to Inclusion (3 items).

Building on the exploratory findings, confirmatory factor analyses (CFA) were conducted to evaluate the factorial validity of the finalized ISA-PS. Alternative measurement models—including a correlated four-factor model, a second-order model, and a bifactor model—were tested to examine whether attitudes toward inclusive sport are best represented by a general latent construct alongside domain-specific factors. CFA results were used to identify the most parsimonious and theoretically coherent representation of the scale's latent structure. The final Hungarian version of the ISA-PS is provided in Supplementary S1.

Finally, an English version of the finalized ISA-PS was produced following the cross-cultural adaptation procedure proposed by Beaton et al. (57). The Hungarian version was independently translated into English by two translators and back-translated into Hungarian by two native English speakers. All versions were reviewed by a five-member expert committee, and the final English version was established by consensus (see Supplementary S1). The English version is provided to facilitate future cross-cultural adaptation and validation and has not yet undergone psychometric validation in English-speaking populations.

### 2.2 Additional components of the test battery

In addition to the ISA-PS, several measures were included to examine associations with theoretically relevant constructs and participant characteristics.

*Introductory questionnaire:* a brief questionnaire was administered to collect sociodemographic information and educational background data, as well as to assess selected inclusion-related experiences, such as participation in inclusion-related coursework, contact experiences with persons with disabilities, or practical experience in inclusive settings.

*Bogardus Scale:* the framework of Bogardus' social distance scale (58) was applied. For nine disability groups (e.g., a person with a physical disability, a person with a visual impairment, a person with an intellectual disability), students were asked to indicate the closest type of relationship they could imagine with the person concerned. Relationship categories were adapted to the student sample as follows: I would accept the person as my partner (7); as a friend (6); as a roommate (5); as a fellow student/colleague (4); as a neighbor (3); as a resident of my settlement (2); I would not live in the same country as the person (1). The measure was included to examine the relationship between attitudes toward inclusive sport practices and social distance toward persons with disabilities. In the present sample, the scale demonstrated excellent internal consistency, with Cronbach's alpha and McDonald's omega coefficients of 0.90.

*Intention-Related Measures*: based on Ajzen's Theory of Planned Behavior (40) and guidelines for the development of theory-based measures (54), additional items representing subjective norms, perceived behavioral control, and intention toward inclusive sport practices were developed using findings from the qualitative phase. However, only the intention item demonstrated satisfactory psychometric characteristics and was therefore retained for subsequent analyses. This statement, rated on a 6-point Likert scale, was included to examine the association between attitudes toward inclusive sport and behavioral intentions.

*Social Dominance Orientation Scale*: the Hungarian-adapted version (59) of the SDO7 scale developed by Ho et al. (60) was administered. The scale includes two subscales, each comprising eight items, assessing acceptance of group-based dominance and anti-egalitarianism. The measure was included to examine the relationship between inclusive sport attitudes and broader preferences for social hierarchy and group inequality. The SDO7 showed excellent internal consistency reliability in the present sample (*α* = 0.92, *ω* = 0.91). Reliability was also good for both the Dominance (*α* = 0.87, *ω* = 0.86) and Anti-egalitarianism (*α* = 0.87, *ω* = 0.86) subscales.

### 2.3 Participants and procedure

Data collection was conducted between April 4 and June 15, 2025. Two independent samples of university students enrolled in sport-related higher education programs were recruited from 9 of the 11 institutions offering sport professional training programs in Hungary. Two institutions did not respond to requests for participation approval. Participation was voluntary and anonymous, and data were collected via the Qualtrics online platform. Questionnaires were distributed during higher education courses using QR codes or direct links. Ethical approval was obtained from the Research Ethics Committee of the Faculty of Education and Psychology at Eötvös Loránd University (approval number: 2025/238).

The first sample was used for exploratory analyses, whereas the second sample formed the basis for confirmatory analyses and validation procedures. Associations between ISA-PS scores and selected inclusion-related experiences, including participation in inclusion-related coursework and contact experiences with persons with disabilities, were also examined within the confirmatory sample to provide further evidence regarding the applicability of the instrument.

#### 2.3.1 Study 1 (exploratory sample)

The questionnaire was completed by 397 students from six universities. After excluding incomplete responses (*n* = 85), the final sample comprised 312 participants (M age = 22.02 ± 3.53 years, range = 18–50). The sample included both male and female students, participants from all years of study, and students enrolled in full-time and part-time sport-related higher education programs. Prospective physical education teachers, recreation and lifestyle students, sports management students and coaches were all represented. In addition, nearly half of the participants reported current or previous participation in inclusion-related coursework, providing variability in inclusion-related educational experiences. Detailed demographic, educational, and inclusion-related characteristics of the participants are presented in Table 1.

**Table 1:** Demographic, educational, and inclusion-related characteristics of Study 1 participants (*n* = 312).

Characteristic	*n*	%
Gender		
Male	177	56.7
Female	135	43.3
University		
Eötvös Loránd University, Budapest	111	35.6
Eötvös Loránd University, Szombathely	91	29.2
Eszterházy Károly Catholic University	14	4.5
Hungarian University of Sports Science	32	10.3
University of Nyíregyháza	13	4.2
University of Pécs	35	11.2
Széchenyi István University	16	5.1
Major		
PE teacher	115	36.9
Coach	47	15.1
Recreation and lifestyle	83	26.6
Sports management	67	21.5
Department		
Full-time	274	87.8
Part-time	38	12.2
Study year		
First year	68	21.8
Second year	97	31.1
Third year	83	26.6
Fourth year	28	9.0
Fifth year	16	5.1
Sixth year or above	20	6.4
Training experience		
Does not lead training	161	51.6
Leads training	135	43.3
Leads training including participants with SEN	16	5.1
Participation in inclusion-related coursework		
Currently not and was not before	164	52.6
Currently not, but was in the past	81	26.0
Currently, but was not before	32	10.3
Currently and was before	35	11.2
Number of inclusion-related courses (*n* = 148)		
One course	96	30.8
Two or more courses	52	16.7
Course type (*n* = 148)		
Optional	16	5.1
Mandatory	132	42.3
Contact with persons with disabilities during coursework (*n* = 147)		
No contact	55	17.6
One occasion	39	12.5
Several occasions	53	17.0
Practical assignment during coursework (*n* = 148)		
No	35	11.2
Yes	113	36.2
Attitudes toward persons with disabilities addressed during coursework (*n* = 148)		
No	16	12.1
Yes	132	42.3

*n* = 312 (100%), the sample contains no missing data (except contact with persons with disabilities during coursework: 1 student) and no extreme outliers.

PE, physical education; SEN, special educational needs.

#### 2.3.2 Study 2 (confirmatory sample)

The questionnaire was completed by 217 students from eight universities. After excluding incomplete responses and cases indicating careless responding (*n* = 50), the final sample comprised 167 participants (M age = 25.40 ± 9.08 years, range = 18–57). The sample included students from a broad range of sport-related study programs, listed here in descending order of frequency: Coaching, Sports Management, Recreation and Lifestyle, Physical Education Teacher Education, and other sport-related programs. Participants represented both full-time and part-time study formats and all years of study. Nearly half of the sample reported current or previous participation in inclusion-related coursework, and among these students, contact experiences with persons with disabilities and practical course activities were common. Detailed demographic, educational, and inclusion-related characteristics of the participants are presented in Table 2.

**Table 2:** Demographic, educational, and inclusion-related characteristics of Study 2 participants (*n* = 167).

Characteristic	*n*	%
Gender		
Male	100	59.9
Female	65	38.9
Prefer not to answer	2	1.2
University		
University of Debrecen	11	6.6
Eötvös Loránd University, Budapest	18	10.8
Eötvös Loránd University, Szombathely	1	0.6
Eszterházy Károly Catholic University	29	17.4
Hungarian University of Sports Science	44	26.3
University of Nyíregyháza	29	17.4
University of Sopron	4	2.4
Széchenyi István University	1	0.6
University of Szeged	30	18.0
Major		
PE teacher	20	12.0
Coach	69	41.3
Recreation and lifestyle	21	12.6
Sports management	54	32.3
Other	3	1.8
Department		
Full-time	114	68.3
Part-time	53	31.7
Study year		
First year	73	43.7
Second year	46	27.5
Third year	27	16.2
Fourth year	19	11.4
Fifth year	1	0.6
Sixth year or above	1	0.6
Training experience		
Does not lead training	75	44.9
Leads training	82	49.1
Leads training including participants with SEN	10	6.0
Participation in inclusion-related coursework		
Currently not and was not before	90	53.9
Currently not, but was in the past	25	15.0
Currently, but was not before	39	23.4
Currently and was before	13	7.8
Number of inclusion-related courses (*n* = 77)		
One course	62	37.1
Two or more courses	15	9.0
Course type (*n* = 77)		
Optional	8	4.8
Mandatory	69	41.3
Contact with persons with disabilities during coursework (*n* = 77)		
No contact	24	14.4
One occasion	20	12.0
Several occasions	33	19.8
Practical assignment involving persons with disabilities during coursework (*n* = 77)		
No	28	16.8
Yes	49	29.3
Attitudes toward persons with disabilities addressed during coursework (*n* = 77)		
No	12	7.2
Yes	65	38.9

*N* = 167. The sample contained no missing data and no extreme outliers.

PE, physical education; SEN, special educational needs.

### 2.4 Statistical analysis

Analyses were conducted sequentially across the two independent samples. Exploratory analyses were performed in Study 1, whereas confirmatory, validity, and background-variable analyses were conducted in Study 2.

Following descriptive analyses (means and standard deviations), univariate normality was assessed using skewness and kurtosis indices (61). All measures showed approximately normal distributions, except for the Social Dominance Orientation Scale (SDO7) and its subscales, which exhibited elevated kurtosis values.

To explore the latent structure of the ISA-PS, an exploratory factor analytic approach was first applied using principal component analysis (PCA) with varimax rotation and Kaiser normalization. Varimax rotation was used to enhance interpretability by maximizing the variance of factor loadings (62). Sampling adequacy was assessed with the Kaiser–Meyer–Olkin (KMO) measure and Bartlett's test of sphericity; KMO values above 0.80 indicated good, and values above 0.90 excellent adequacy, while a significant Bartlett's test confirmed suitability for factor analysis (63, 64).

Factor retention was guided by eigenvalues greater than 1.00 and a conservative loading threshold of 0.50 to ensure substantial item–factor associations (65). Competing solutions were evaluated, and the final structure was selected based on both statistical criteria and theoretical interpretability (66). Internal consistency of the total scale and subscales was examined using Cronbach's alpha coefficients, with values ≥0.70 considered acceptable and values ≥0.80 indicating good reliability (65, 67). Item–total correlations were inspected, with coefficients ≥0.30 regarded as satisfactory.

Building on the exploratory results, confirmatory factor analyses (CFA) were conducted to test alternative measurement models, including a correlated four-factor model, a second-order model, and a bifactor model. Given the ordinal nature of the items and violations of multivariate normality, models were estimated using the robust weighted least squares estimator with mean and variance adjustment (WLSMV), which is appropriate under these conditions (68, 69). Model fit was evaluated using the robust *χ*^2^ statistic and multiple fit indices: the Comparative Fit Index (CFI), Tucker–Lewis Index (TLI), Root Mean Square Error of Approximation (RMSEA) with 90% confidence intervals, and the Standardized Root Mean Square Residual (SRMR). CFI and TLI values ≥0.90 indicated acceptable fit and ≥0.95 excellent fit; RMSEA values ≤0.08 suggested acceptable fit and ≤0.06 good fit; SRMR values ≤0.08 were considered acceptable (70–72).

In the bifactor model, each item was specified to load on a general factor representing overall inclusive sport attitudes, as well as on one orthogonal specific factor capturing domain-specific variance, consistent with bifactor modeling conventions (73, 74). Standardized factor loadings ≥0.40 were interpreted as substantively meaningful (75). Model comparisons were based on overall fit indices and theoretical coherence rather than *χ*^2^ difference testing, due to the sensitivity of the *χ*^2^ statistic to sample size (76).

Construct validity was examined through both internal and external convergent and discriminant validity analyses. Internal convergent validity was assessed within the CFA framework using average variance extracted (AVE) and composite reliability (CR), which were calculated from the standardized CFA parameter estimates (i.e., factor loadings and residual variances). CR values ≥ 0.70 were considered indicative of adequate reliability, while AVE values ≥ 0.50 were interpreted as evidence of convergent validity; lower AVE values were regarded as acceptable when accompanied by sufficient CR, particularly in the context of bifactor models, where variance may be distributed across both general and specific factors (75, 77). Internal discriminant validity was evaluated using the Fornell–Larcker criterion by comparing AVE values with squared correlations between latent constructs. External construct validity was assessed by examining associations with theoretically related and unrelated constructs. Pearson correlations were used for approximately normally distributed variables and Kendall's *τ* when distributional assumptions were violated (e.g., for SDO7 variables). Strong correlations (*r* ≥ 0.50) among theoretically related constructs supported convergent validity, whereas weak or non-significant correlations (*r* < 0.30) with distinct constructs indicated discriminant validity (69, 78). Following established recommendations, external validity was further evaluated based on the magnitude and direction of associations with conceptually related and unrelated measures, interpreted according to Cohen's (79) effect size guidelines.

As an additional source of validity evidence, associations between ISA-PS scores and selected inclusion-related experiences were also examined. For these analyses, assumptions of parametric procedures were evaluated using the same criteria described above. Depending on variable characteristics and distributional assumptions, both parametric and non-parametric statistical procedures were applied. Group differences were analyzed using independent-samples t tests or one-way analysis of variance (ANOVA), with Mann–Whitney U and Kruskal–Wallis H tests applied when assumptions were violated (80). Effect sizes were calculated for all analyses and interpreted according to Cohen's (79) guidelines.

Exploratory analyses were conducted using IBM SPSS Statistics 30.0. Confirmatory factor analysis (CFA) was performed using the Structural Equation Modeling (SEM) module in JASP (version 0.95.4). Statistical significance was set at *α* = 0.05.

## 3 Results

### 3.1 Study 1: exploratory analyses

#### 3.1.1 Exploratory factor structure of the ISA-PS

An exploratory factor analytic approach using principal component extraction with varimax rotation (*n* = 312) was employed to examine the underlying structure of the Inclusive Sport Attitudes Scale for Pre-Service Sport Professionals (ISA-PS). Sampling adequacy was supported by a high Kaiser–Meyer–Olkin (KMO) value (0.93) and a significant Bartlett's test of sphericity (*p* < 0.001), indicating that the data were suitable for factor analysis.

The initial unrestricted solution suggested five components based on eigenvalues greater than one. Following iterative item refinement based on factor loadings, cross-loadings, conceptual interpretability, and the predefined criteria of the scale development process, alternative two-, three-, and four-factor solutions were examined. Considering both statistical criteria and theoretical interpretability, a four-factor solution was retained (see Table 3 for factor loadings and Table 4 for descriptive statistics). The final model consisted of 16 items loading on four factors: Positive Willingness (PW), Negative Components for Sport Professionals (NC), Positive Components for Participants (PC), and Barriers to Inclusion (BI). All retained items demonstrated substantial factor loadings, and the four factors jointly accounted for 65.2% of the total variance, supporting the proposed factorial structure of the ISA-PS.

**Table 3:** PCA factor loadings (varimax rotation), communalities and explained variance of the ISA-PS (*n* = 312).

Item	PW (F1)	NC (F2)	PC (F3)	BI (F4)	h^2^
8	0.81	0.10	0.15	−0.01	0.69
12	0.81	0.04	0.16	−0.05	0.68
9	0.81	0.18	0.16	−0.15	0.73
16	0.78	0.26	0.23	0.05	0.73
13	0.71	0.10	0.15	0.08	0.55
11 (R)	0.15	0.84	0.11	0.10	0.75
7 (R)	0.11	0.83	0.15	0.12	0.73
10 (R)	0.17	0.77	−0.01	0.12	0.64
14 (R)	−0.03	0.70	0.03	−0.02	0.49
15 (R)	0.38	0.64	0.03	0.15	0.57
5	0.24	0.09	0.80	−0.10	0.71
4	0.18	0.06	0.80	−0.17	0.70
1	0.21	0.09	0.73	−0.01	0.59
3 (R)	−0.06	0.07	−0.11	0.80	0.66
6 (R)	−0.11	0.04	−0.25	0.75	0.65
2 (R)	0.16	0.34	0.14	0.63	0.55
Variance^e^ (%)	21.6	19.6	13.1	10.8	
Variance^c^ (%)	21.6	41.2	54.3	65.2	

Principal component analysis with varimax rotation. KMO = 0.86. Bartlett's test of sphericity: *χ*^2^(120) = 2,104.28, *p* < .001.

PW, positive willingness; NC, negative components for sport professionals; PC, positive components for participants; BI, barriers to inclusion; h^2^, communality; Varianceᵉ denotes explained variance and Varianceᶜ denotes cumulative variance. Reverse-coded items are marked with (R). Factor loadings greater than 0.50 are highlighted with gray shading.

**Table 4:** Descriptive statistics for the ISA-PS total scale and subscales in the four-factor model (*n* = 312).

Scale	Subscales	Min	Max	M	SD	Sk	Ku	*α*	*ω*
ISA-PS		16	96	62.64	10.78	0.241	0.543	0.83	–
	PW	5	30	20.33	4.98	−0.312	0.567	0.87	0.87
	NC	5	30	19.63	5.05	0.141	−0.142	0.84	0.84
	PC	3	18	13.87	2.99	−0.702	0.244	0.75	0.75
	BI	3	18	8.81	2.78	0.567	0.455	0.61	0.62

M, mean; SD, standard deviation; Sk, skewness; Ku, kurtosis; ISA-PS, inclusive sport attitudes scale for pre-service sport professionals (total scale); PW, positive willingness; NC, negative components for sport professionals; PC, positive components for participants; BI, barriers to inclusion; α, cronbach's alpha; ω, McDonald's omega. McDonald's omega coefficients are reported for the four subscales; Cronbach's alpha is reported for the total scale.

#### 3.1.2 Descriptive statistics and internal consistency

As shown in Table 4, the total ISA-PS scale demonstrated good internal consistency (*α* = 0.83). Subscale reliabilities were satisfactory for PW (*α* = 0.87), NC (*α* = 0.84), PC (*α* = 0.75), and moderate for BI (*α* = 0.61) factors. McDonald's omega coefficients calculated for the four subscales closely corresponded to the respective Cronbach's alpha values (*ω* = 0.62–0.87), providing additional support for the internal consistency of the extracted factor solution. Item communalities ranged from 0.49 to 0.75, indicating that extracted factors accounted for a substantial proportion of item variance (Table 3).

### 3.2 Study 2: confirmatory analyses and validity evidence

#### 3.2.1 Confirmatory factor analysis

Confirmatory factor analysis was conducted using the WLSMV estimation method for ordinal items, due to violations of multivariate normality. Model fit was evaluated using the robust *χ*^2^ test, along with the CFI, TLI, RMSEA, and SRMR fit indices. CFA was conducted on an independent sample (*n* = 167) to evaluate the factor structure of the scale. Sample size adequacy for CFA was supported by Hoelter's Critical N values exceeding recommended thresholds. Four alternative models were compared: a one-factor model, a correlated four-factor model, a second-order factor model, and a bifactor model (Table 5).

**Table 5:** Fit indices for CFA models (*n* = 167).

Model	X2/df	CFI	TLI	SRMR	RMSEA
one-factor	3.261	0.624	0.566	0.130	0.117
four-factor	1.764	0.880	0.853	0.075	0.068
second-order	1.659	0.895	0.873	0.080	0.063
bifactor	1.366	0.948	0.930	0.061	0.047

Fit statistics include *χ*²/df, CFI, TLI, SRMR, RMSEA. Higher CFI and TLI values indicate better fit, whereas lower *χ*²/df, SRMR, and RMSEA values indicate better model fit.

Among the tested models, the bifactor model demonstrated the best overall fit to the data. This model supported the presence of a general factor, representing attitudes toward inclusive sport practices, alongside four specific factors. The ECV value (≈0.47) suggested that variance was distributed across both the general and specific factors.

**Figure 1:**
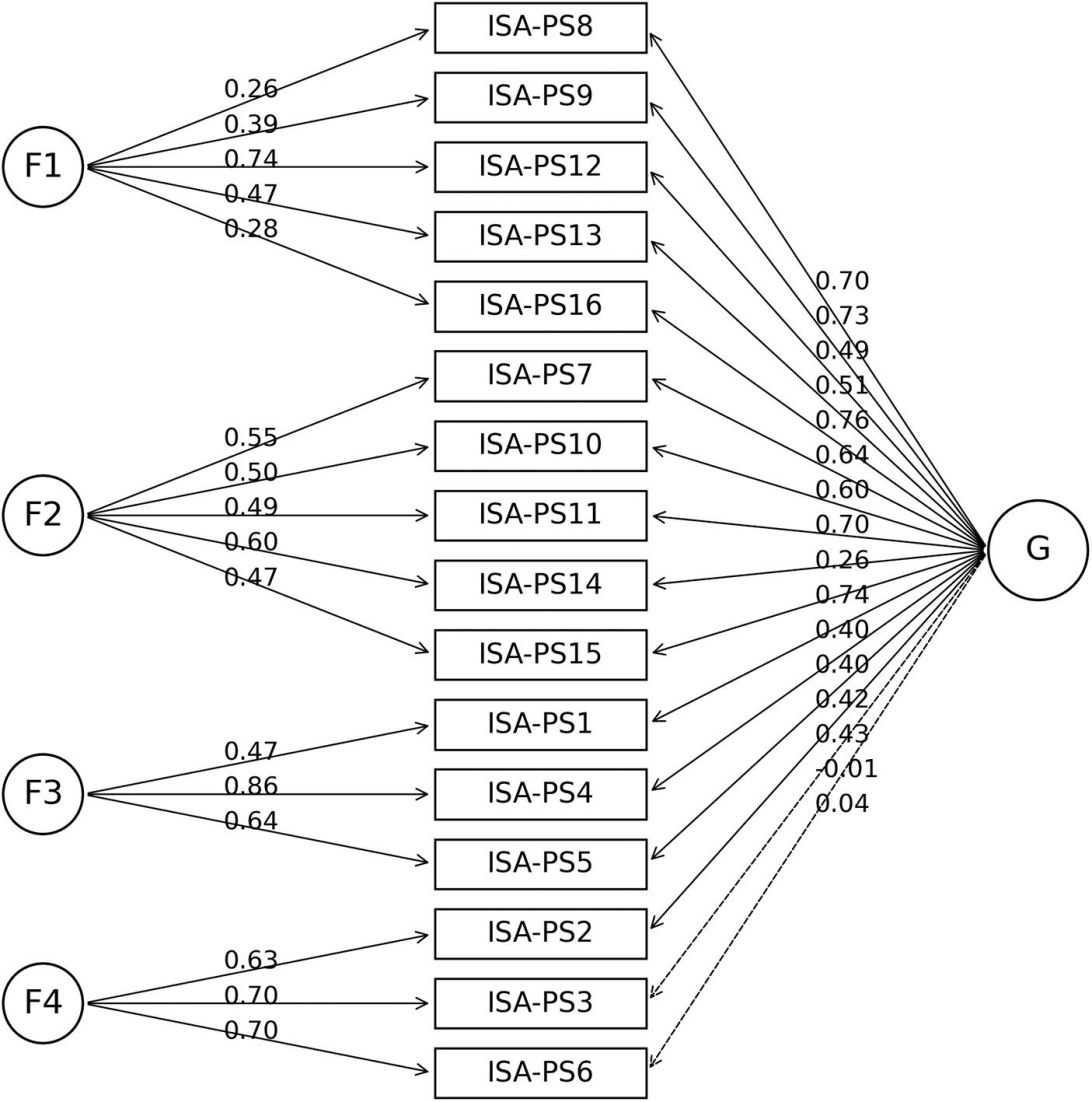
Bifactor confirmatory factor analysis (CFA) model of the ISA-PS scale. The model includes one general factor (G) and four specific factors (F1–F4). Standardized factor loadings are displayed along the arrows. Solid lines indicate statistically significant loadings, whereas dashed lines indicate non-significant loadings. Error terms and inter-factor correlations are omitted for clarity.

Standardized factor loadings in the bifactor model are presented in Figure 1. Loadings on the specific factors were generally moderate to high, while the general factor demonstrated meaningful loadings for most items. Two items (ISA-PS3 and ISA-PS6) did not load significantly on the general factor, suggesting that their variance was primarily captured by the corresponding specific factor.

Taken together, these findings indicate that while the ISA-PS can be meaningfully interpreted at the total score level, the specific factors retain substantial explanatory power beyond the general factor. These findings should, however, be interpreted in light of the methodological considerations discussed in the Discussion and Limitations sections.

#### 3.2.2 Reliability of the ISA-PS bifactor structure

The ISA-PS total scale showed high reliability (*α* = 0.87, *ω* = 0.94). Reliability estimates for the specific factors were more variable, with lower *ω* values observed for some factors (Table 6). This pattern is consistent with bifactor models, in which a substantial proportion of common variance may be captured by the general factor, resulting in reduced reliability estimates for specific factors (74).

**Table 6:** Reliability indices (Cronbach's α and McDonald's ω) for the ISA-PS bifactor model (*n* = 167).

Factor	α	ω
General (G)	0.87	0.72
F1	0.87	0.28
F2	0.88	0.40
F3	0.81	0.59
F4	0.71	0.70
Total	0.87	0.94

G, general factor; F1, positive willingness; F2, negative omponents for sport professionals; F3, positive components for participants; F4, barriers to inclusion; Total, total ISA-PS score; α, Cronbach's alpha; ω, McDonald's omega.

#### 3.2.3 Internal convergent and discriminant validity

Internal convergent validity of the ISA-PS was evaluated using average variance extracted (AVE) and composite reliability (CR) based on standardized estimates from the bifactor CFA model (Table 7).

**Table 7:** Convergent validity and composite reliability of the ISA-PS bifactor model (*n* = 167).

Factor	AVE	CR
Positive Willingness (PW)	0.37	0.71
Negative Components for Sport Professionals (NC)	0.44	0.79
Positive Components for Participants (PC)	0.55	0.77
Barriers to Inclusion (BI)	0.48	0.74
General factor (G)	0.43	0.91

AVE, average variance extracted; CR, composite reliability. Values were calculated based on standardized factor loadings and residual variances from the bifactor CFA model.

As shown in Table 7, CR values exceeded the recommended threshold of 0.70 for all latent constructs, indicating satisfactory internal consistency. AVE values met or exceeded the conventional cutoff of 0.50 for the Positive Components for Participants (PC) factor, while slightly lower values were observed for the remaining constructs. As noted in the methodological literature, AVE values below 0.50 may be acceptable when CR is adequate, particularly in multidimensional and bifactor models (75, 77). Internal discriminant validity was examined using the Fornell–Larcker criterion. Because the specific factors in the final bifactor model were specified as orthogonal, inter-factor correlations were constrained to zero. Consequently, the criterion was met for all specific factors. Overall, these findings provide support for the internal convergent and discriminant validity of the ISA-PS.

#### 3.2.4 External convergent and discriminant validity

Convergent validity was supported by positive associations with the Bogardus Social Distance Scale and the Intention toward Inclusive Sport (IIS) item, with the strongest correlations observed for the PW subscale and the total ISA-PS score.

Discriminant validity was further strengthened by the absence of significant associations between the ISA-PS (total score and subscales) and the Social Dominance Orientation Scale (SDO7), including both the Dominance and Anti-egalitarianism subdimensions. The consistently weak and non-significant correlations indicate that inclusive sport attitudes, as measured by the ISA-PS, represent a construct conceptually distinct from general dominance- and hierarchy-oriented social attitudes (Table 8).

**Table 8:** External validity correlations (*n* = 167).

Measures	PW	NC	PC	BI	ISA-PS
Bogardus Scale	0.35**	0.19*	0.15	0.10	0.31**
IIS	0.78**	0.41**	0.22**	0.21**	0.64**
SDO7 D	−0.06	−0.07	0.05	−0.11	−0.05
SDO7 A	0.08	0.10	0.08	−0.01	0.09
SDO7 full	−0.01	0.02	0.09	−0.06	0.03

PW, positive willingness; NC, negative components for sport professionals; PC, positive components for participants; BI, barriers to inclusion; ISA-PS, inclusive sport attitudes scale for pre-service sport professionals (total scale); IIS, intention toward inclusive sport; SDO7, social dominance orientation scale; SDO7 D, dominance subscale; SDO7 A, anti-egalitarianism subscale.

* *p* < 0.05.

** *p* < 0.001.

#### 3.2.5 Final structure of the ISA-PS

**Figure 2:**
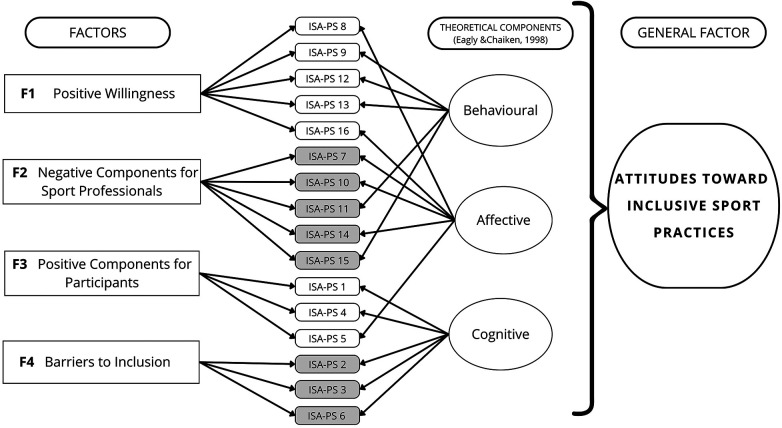
Final structure of the Inclusive Sport Attitudes Scale for Pre-Service Sport Professionals (ISA-PS), integrating the bifactor model and the proposed multidimensional attitude framework. Reverse-coded items are indicated with a dark background.

The final version of the ISA-PS was established based on the combined evidence from scale development, exploratory and confirmatory analyses, and theoretical considerations (Figure 2).

The 16-item bifactor ISA-PS assesses attitudes toward inclusive sport participation by incorporating multiple perspectives, including factors related to both sport professionals and participants. The three attitudinal components (cognitive, affective, and behavioral) are represented across the 16 items in a 5–5–6 distribution. Positively and negatively worded items are alternated to help maintain respondent attention and to reduce the potential for response patterns associated with inattentive or routine responding. Reverse-coded items are indicated in Figure 2.

### 3.3 Associations with inclusion-related educational experiences

To explore the practical applicability of the ISA-PS, additional analyses were conducted examining associations with selected inclusion-related educational experiences, including participation in inclusive sport courses, contact experiences with persons with disabilities during coursework, practical training experiences, and exposure to attitude-shaping course content. Significant differences in ISA-PS scores were observed in relation to inclusive sport course participation and contact experiences with persons with disabilities.

#### 3.3.1 Inclusion-Related coursework

Participants were categorized according to their inclusion-related coursework experience as having (1) no prior or current coursework, (2) previous but no current coursework, (3) current but no previous coursework, or (4) both previous and current coursework. Significant group differences emerged for both the Positive Willingness factor and the total ISA-PS score (Table 9).

**Table 9:** Differences in ISA-PS scores according to inclusion-related educational experiences (*n* = 167).

Coursework experience	No previous or current coursework	Previous only	Current only	Previous and current	F(df)	*p*	*η* ^2^
Positive Willingness	19.60 (5.27)	19.24 (4.97)	21.82 (4.71)	24.92 (4.13)	5.75 (3.163)	<0.001	0.10
ISA-PS total	61.03 (11.69)	60.52 (10.22)	66.62 (12.18)	71.38 (12.21)	4.73 (3.163)	0.003	0.08

Contact frequency during coursework	No contact	One occasion	Multiple occasions	F(df)	*p*	*η* ^2^
Positive Willingness	21.92 (3.98)	18.85 (5.51)	22.82 (4.94)	4.34 (2.74)	0.016	0.11
ISA-PS total	65.21 (12.42)	59.45 (10.92)	69.24 (11.24)	4.49 (2.74)	0.014	0.11

Values represent means with standard deviations in parentheses.

ISA-PS, inclusive sport attitudes scale for pre-service sport professionals; *η*^2^, eta squared.

Significant group differences emerged for both the Positive Willingness factor and the total ISA-PS score (Table 9). For Positive Willingness, the effect was of moderate magnitude, F(3,163) = 5.75, *p* < .001, *η*^2^ = .10. *post hoc* comparisons indicated that students with both previous and current coursework scored significantly higher than those with no coursework experience (*p* = .003, d = 1.05) and those with only previous coursework experience (*p* = .007, d = 1.18).

**Figure 3:**
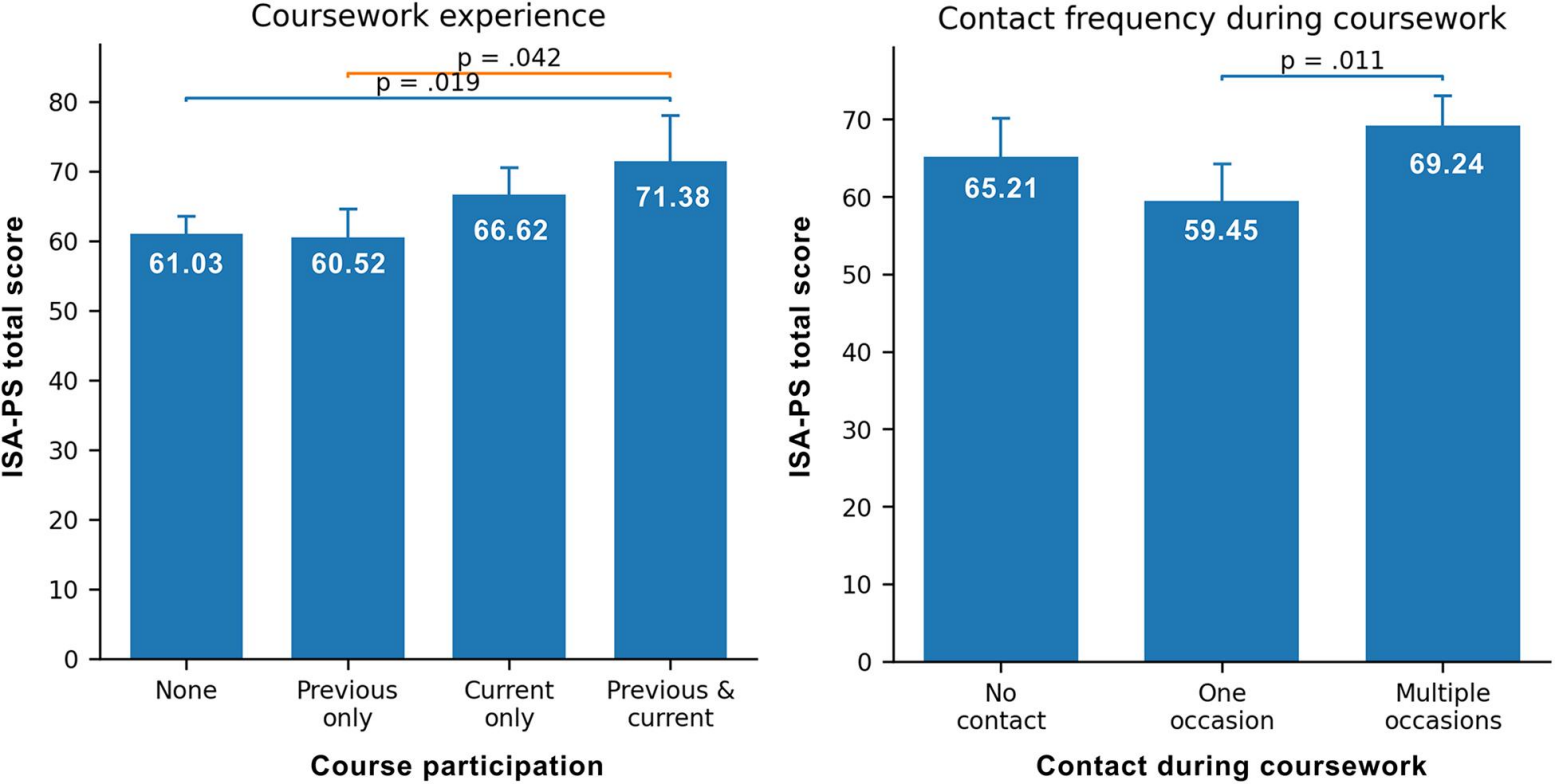
Associations between inclusion-related educational experiences and attitudes toward inclusive sport practices. Mean ISA-PS total scores are presented according to coursework experience and frequency of contact with persons with disabilities during coursework. Error bars represent 95% confidence intervals. Significant pairwise differences for the total ISA-PS score identified in *post hoc* analyses are indicated above the bars. ISA-PS, inclusive sport attitudes scale for pre-service sport professionals.

A similar pattern was observed for the total ISA-PS score, F(3,163) = 4.73, *p* = .003, *η*^2^ = .08. Students with both previous and current coursework experience obtained significantly higher ISA-PS scores than students with no coursework experience (*p* = .019, d = 0.87) and those who had participated only in the past (*p* = .042, d = 0.94). No significant differences were observed for the remaining factors. The pattern observed for the total ISA-PS score is illustrated in Figure 3.

#### 3.3.2 Contact experiences with persons with disabilities

Among students who had participated in inclusion-related coursework (*n* = 77), ISA-PS scores were compared according to the frequency of personal contact with persons with disabilities during the course. Participants were classified into three groups: (1) no contact, (2) one contact occasion, and (3) multiple contact occasions.

Significant group differences were observed for both Positive Willingness and the total ISA-PS score (Table 9). For Positive Willingness, a moderate effect emerged, F(2,74) = 4.34, *p* = .016, *η*^2^ = .11. Students reporting multiple contact experiences obtained significantly higher ISA-PS scores than those reporting a single contact experience (*p* = .015).

A similar pattern was found for the total ISA-PS score, F(2,74) = 4.49, *p* = .014, *η*^2^ = .11. Students reporting multiple contact experiences scored significantly higher on the ISA-PS total scale than those reporting a single contact experience (*p* = .011). No significant differences were found for the remaining factors. Results are presented in Figure 3. Given the exploratory nature of these analyses and the relatively small subgroup sample sizes, these findings should be interpreted with appropriate caution.

## 4 Discussion

The growing demand for sport professionals with appropriate inclusive attitudes and training, together with the increasing presence of inclusion-related content in sport professional education, highlights the need for valid instruments capable of assessing attitudes toward inclusive sport practices across diverse sport-related educational contexts. Building on existing multidimensional instruments, the ISA-PS was developed to extend the assessment of inclusive sport attitudes beyond teacher education to a broader range of sport-related educational programs. Its development was based on a systematic review of the literature, expert evaluation, and a qualitative pilot study, and was theoretically informed by a multidimensional conceptualization of attitudes (35) and Ajzen's Theory of Planned Behavior (40). Exploratory analyses supported a four-factor structure, which was subsequently examined in an independent sample using confirmatory factor analysis.

Among the tested solutions, a bifactor model demonstrated the best fit, supporting the presence of a general factor representing attitudes toward inclusive sport practices alongside four specific factors. Reliability and validity analyses provided further support for the psychometric adequacy of the ISA-PS. Overall, the findings suggest that the ISA-PS may be considered a theoretically grounded, reliable, and valid instrument for assessing attitudes toward inclusive sport practices at both the total-score and specific-factor levels.

The theoretical framework of the ISA-PS was grounded in a multidimensional conceptualization of attitudes. Several internationally used instruments—such as the ATIPDPE-R (48), *Attitudes Towards Inclusive Physical Education Scale* (ATIPE) (81), and PEATID-III (43)—primarily focus on the cognitive component of attitudes. In developing the ISA-PS, we sought to capture attitudes toward inclusive sport practices using a multidimensional approach that reflects the complexity of the construct. Whereas multidimensional instruments typically organize their factor structure along the core components of attitudes (e.g., MATIES and ISHST) (46, 47), the ISA-PS represents these components through factors reflecting different aspects of inclusive sport practices.

As the ISA-PS was theoretically informed by Ajzen's TPB (40), constructs related to subjective norms and perceived behavioral control were also assessed, but could not be retained for further analyses due to insufficient psychometric performance. This finding may suggest that, within the present sample, attitudes toward inclusive sport were more readily measurable than other TPB components. This pattern may partly reflect the limited practical experience of many participants, which may have made perceptions of behavioral control in inclusive sport settings more difficult to evaluate. Intention toward inclusive sport was therefore examined separately through an additional item, while the ISA-PS itself ultimately focused on the attitudinal component of the TPB framework, similar to the widely used MATIES (46).

Principal component analysis supported a four-factor structure of the ISA-PS, accounting for 65.2% of the total variance This value approaches or exceeds those reported for comparable instruments in the international literature, including MATIES, 70.6% (46); ATIPDPE-R, 65.1% (48); TAIS, 49.1% (45); and SACIE-R, 47.3% (44). Factor loadings for the ISA-PS ranged from 0.63 to 0.84, which can be considered robust in an international comparison (MATIES: 0.52–0.90; ATIPDPE-R: 0.71–0.91), suggesting that the scale captures well-differentiated yet internally coherent factors.

Cronbach's alpha values for the ISA-PS subscales (0.87, 0.84, 0.75, and 0.61) were largely comparable to those reported for previously used instruments, particularly for the first three subscales. Reported reliability coefficients for the SACIE-R range from *α* = 0.70 to 0.86 (44), while values between *α* = 0.78 and 0.91 have been reported for the MATIES (46). The Barriers to Inclusion factor demonstrated comparatively weaker reliability than those of the remaining subscales. Although methodological literature suggests that such values may be acceptable in exploratory attitude research, particularly for short scales assessing heterogeneous content domains (82–84), the independent interpretation of this factor should currently be approached with greater caution until additional validation studies provide further evidence regarding its stability and measurement properties. Internal consistency for the total scale was found to be adequate (*α* = 0.83), indicating that the items collectively contribute in a consistent manner to the measurement of the target construct.

Building on the exploratory findings, confirmatory factor analysis was conducted to further examine the proposed factor structure. Based on fit indices meeting internationally accepted cutoff criteria, a bifactor model demonstrated the best fit (CFI = 0.948; TLI = 0.930; RMSEA = 0.047; SRMR = 0.061). In this model, four specific factors were retained alongside a general factor representing attitudes toward inclusive sport practices. In contrast, previous confirmatory studies of inclusion-related attitude scales have generally supported either correlated multifactor structures, such as the three-factor SACIE-R (85), or unidimensional models, such as the TAIS (45). These models are well suited for capturing general attitudes toward inclusion and have proven useful in educational research; however, they primarily emphasize overall evaluative tendencies rather than the simultaneous consideration of general dispositions and thematically differentiated attitude aspects.

This dual representation of general and specific attitude dimensions was further supported by the explained common variance (ECV ≈ 0.47), indicating that the general factor accounts for a substantial, though not exclusive, proportion of the shared variance among items. This pattern suggests that, alongside an overarching attitudinal construct, specific thematic factors also play a meaningful role within the measurement structure. The presence of an interpretable general factor supports the use of the ISA-PS total score in the evaluation of educational and learning processes, while the retained specific factors may facilitate a more nuanced understanding of the underlying attitudinal structure. This distinction is particularly relevant in the context of pre-service sport professionals, for whom positive inclusive intentions may coexist with perceived practical constraints that could influence future professional behavior.

Consistent with this interpretation, factor loadings showed greater variability in the bifactor model than in the exploratory solution, reflecting the partitioning of variance between general and specific factors. Nevertheless, reliability estimates remained acceptable, and subscale Cronbach's alpha coefficients were slightly higher than those observed in the exploratory phase. Hierarchical omega coefficients calculated within the bifactor model indicate that the ISA-PS total score can be interpreted with a high level of reliability (*ω* = 0.94), while the general factor accounts for a substantial, though not exclusive, proportion of the variance in item responses (*ω*_h_ = 0.72). The independent contribution of the specific factors varies in magnitude (*ω*_h_s = 0.28–0.70), which is consistent with the complexity of the construct and the presence of thematically distinct content domains. Internal convergent validity was further supported by adequate to high composite reliability values across all factors. Although AVE values for several factors remained below the conventional 0.50 threshold, this pattern is not uncommon in multidimensional and bifactor models and may be considered acceptable when composite reliability is satisfactory. Overall, these findings provide additional support for the internal construct validity of the ISA-PS and the interpretation of the instrument at both the total-score and specific-factor levels. However, these findings should be interpreted with appropriate caution, as the confirmatory sample was relatively modest and no cross-validation or test–retest analyses were conducted. During the assessment of external convergent validity, the total ISA-PS score showed a moderate positive correlation with the disability-adapted version of the Bogardus Social Distance Scale (*r* = 0.31). At the subscale level, associations were generally weak or non-significant (*r* = 0.10–0.35), which may be attributable to the fact that the Bogardus scale assesses general attitudes toward disability and does not specifically reflect the context of sport and inclusion. Nevertheless, the use of the Bogardus scale was justified by the lack of a validated Hungarian-language attitude measure more closely aligned in content with inclusive sport practices. The association between the ISA-PS and intention toward inclusive sport (IIS) was moderate to strong (*r* = 0.64), which is consistent with theoretical models linking attitudes to behavioral intention (38, 86, 87), and further supports the addition of the intention-related item alongside the 16 ISA-PS items.

At the same time, because the present evidence is limited to cross-sectional associations with social distance and behavioral intention, further research incorporating behavioral indicators and longitudinal designs is needed to more directly evaluate the practical applicability of the ISA-PS.

External discriminant validity was assessed through comparisons with the Social Dominance Orientation Scale (SDO7) and its Dominance and Anti-egalitarianism subscales. The ISA-PS total score and subscales showed weak or non-significant associations with SDO7 indices. These findings indicate that attitudes toward inclusive sport practices are empirically distinct from general dominance- and hierarchy-oriented social attitudes, thereby supporting the discriminant validity of the scale. Although the absence of associations primarily supports discriminant validity, it may also indicate that attitudes toward inclusive sport are more strongly related to context-specific professional beliefs than to general ideological orientations. Furthermore, attitudes toward disability may not necessarily follow the same patterns observed for other social groups commonly examined in social dominance research, as disability is often associated with more paternalistic forms of evaluation (88) than with overt intergroup competition or hierarchy.

Additional validity evidence was provided by the associations between ISA-PS scores and inclusion-related educational experiences. Identifying the factors influencing attitudes toward inclusion is particularly important, as sport professionals' attitudes may influence the successful delivery of inclusive sport. However, the educational benefits of inclusive practices extend beyond sport and physical education. Research conducted across school and higher education settings has shown that inclusive educational environments may foster students' social skills, such as empathy and cooperation, while also promoting higher self-esteem, supporting identity development, and contributing to academic achievement (89, 90).

Students with continuous coursework experience demonstrated higher scores on both the Positive Willingness factor and the total ISA-PS scale than students with no coursework experience or only previous coursework experience. Although the cross-sectional design does not allow causal conclusions, this pattern may suggest that sustained engagement with inclusion-related content is associated with more favorable attitudes toward inclusive sport. At the same time, the possibility of self-selection cannot be excluded, as students with more positive initial attitudes may be more likely to participate in additional inclusion-related courses. Previous research has reported inconsistent findings regarding the relationship between coursework and inclusion-related attitudes. Some studies have found more favorable attitudes among participants with relevant coursework experience (22, 91, 92), whereas others have reported no significant differences associated with coursework participation (93–95).

A similar pattern emerged for contact experiences with persons with disabilities during coursework. Significant differences were observed for both the total ISA-PS score and the Positive Willingness factor. Students reporting multiple contact experiences demonstrated more favorable attitudes than those reporting only a single contact experience. Previous studies have primarily focused on the role of pre-existing personal relationships with persons with disabilities in shaping professional attitudes toward inclusion (85, 96, 97), whereas research examining structured contact opportunities within educational settings and inclusive sport events has generally reported positive effects on inclusion-related attitudes (98, 99). Importantly, the difference emerged between single and repeated contact experiences rather than between contact and no-contact groups, suggesting that the frequency of contact may be more relevant than the mere occurrence of contact. This interpretation is consistent with both Zajonc's (100) mere exposure effect and Allport's (101) contact hypothesis. Whereas the mere exposure perspective suggests that repeated encounters may enhance familiarity and positive evaluations, contact theory emphasizes that attitudinal change is most likely when interactions occur under favorable and sustained conditions. However, the present study did not assess the quality, intensity, or context of these interactions; therefore, it remains unclear which characteristics of contact experiences contribute most strongly to the development of more favorable attitudes.

Notably, significant differences emerged not only for the total ISA-PS score but also for the Positive Willingness factor, whereas no significant group differences were observed for the remaining specific factors. This pattern suggests that inclusion-related educational experiences may be more strongly associated with participants' willingness to engage in inclusive sport than with other specific aspects of inclusive sport attitudes. The presence of significant differences at both the total-score and factor levels also supports the practical value of interpreting the ISA-PS within a bifactor framework, although these exploratory findings should be interpreted with caution given the relatively small and unequal subgroup sizes.

### 4.1 Limitations

Several limitations should be considered when interpreting the findings of the present study and the proposed ISA-PS instrument. This study relied exclusively on self-report measures, which may be influenced by social desirability, particularly given the normative expectations surrounding inclusion in educational and sport-related contexts. Participation was voluntary, which may have introduced self-selection bias and should be considered when interpreting the generalizability of the findings. In addition, the cross-sectional design and the absence of test–retest assessment do not allow conclusions regarding the temporal stability of the ISA-PS or potential changes in attitudes over the course of professional training, which is particularly relevant in pre-service populations.

Furthermore, the data were collected within a single national higher education context; therefore, the findings cannot be readily generalized to other countries, educational systems, or cultural settings without further cross-national validation. In addition, the composition of the exploratory and confirmatory samples differed with regard to the distribution of study programs, with physical education teacher students representing the largest subgroup in the exploratory sample, whereas coach and sport management students constituted the largest groups in the confirmatory sample. Although the factor structure remained stable across the two phases, this difference should be considered when interpreting the generalizability of the findings.

While the ISA-PS was developed within a Theory of Planned Behavior framework, the present study was ultimately limited to the assessment of attitudes toward inclusive sport and their association with behavioral intention. Constructs related to subjective norms and perceived behavioral control could not be evaluated further due to insufficient psychometric performance.

From a psychometric perspective, the confirmatory sample size was close to the lower end of commonly recommended ranges for evaluating bifactor models, and the proposed factor structure was not examined through cross-validation in an additional independent sample. These methodological considerations should be taken into account when interpreting the psychometric findings. Although the four-factor structure was supported overall, the Barriers to Inclusion factor demonstrated weaker indicators in the exploratory phase than the remaining factors. Its performance improved in the confirmatory analyses; however, the mixed psychometric findings across the exploratory and confirmatory phases suggest that further refinement and validation of this factor are warranted to determine the stability and practical usefulness of a standalone score.

Although external validity was supported through associations with social distance and behavioral intention, the present evidence remains limited to cross-sectional associations with these related constructs. Behavioral indicators of inclusive sport practice were not assessed, no dedicated measure of social desirability was included, and longitudinal data were not available. These considerations should be taken into account when interpreting the practical applicability of the ISA-PS.

Several limitations should also be considered when interpreting the findings related to educational experiences. The observed associations were not replicated in an independent sample and may partly reflect characteristics of the present study population. In addition, some subgroup comparisons involved relatively small and unequal group sizes, which may have reduced statistical power. The cross-sectional design further limits causal interpretation of the observed associations. The educational variables were assessed using relatively broad and predominantly dichotomous categories, limiting the examination of the content, quality, intensity, and implementation of the reported experiences. This limitation may also help explain the absence of significant associations for practical components and attitude-shaping activities, as the underlying concepts may not have been interpreted consistently across participants.

Finally, although external validity was supported through associations with related constructs, the study did not assess either the actual implementation of inclusive sport practices or the specific content and quality of the educational experiences reported by participants. Consequently, the findings should be interpreted primarily at the level of attitudes and self-reported educational experiences rather than observable professional behavior or verified training effects.

### 4.2 Future research

Taken together, the present findings and limitations identify several directions for future research. A key priority for future research is the international validation of the ISA-PS. Validation of the English version, followed by adaptation and validation in additional languages, would provide opportunities for cross-cultural comparisons and for examining the generalizability of the instrument across diverse educational and cultural contexts. Alongside international validation, an important step would be to examine the stability and practical relevance of the bifactor structure, including through cross-validation in additional independent samples and test–retest assessment, with particular attention to the contribution of the specific factors beyond the general attitude dimension. Further research should also focus on the continued refinement and validation of the Barriers to Inclusion factor. Longitudinal designs, including test–retest assessments, would allow the investigation of attitudinal stability and change across different stages of professional training. In addition, the development and validation of a version for practicing sport professionals may facilitate the examination of inclusive sport attitudes beyond the pre-service stage and enable comparisons across different phases of professional development.

Beyond psychometric validation, future studies should seek to clarify the educational mechanisms associated with attitudes toward inclusive sport practices. In particular, intervention studies targeting specific courses or training modules, complemented by observational or practice-based indicators, may help identify how attitudinal components translate into inclusive professional behavior.

### 4.3 Practical implications

Across sport professional education programs, the ISA-PS may be used to monitor students' attitudinal profiles across different stages of training and to identify areas of potential uncertainty or resistance related to inclusive practice. The instrument may also be applied in pre–post evaluations of inclusion-focused coursework or other educational interventions, thereby offering supplementary information for program evaluation. When complemented by observational measures, analyses of course characteristics, or controlled evaluations of different inclusion-focused educational strategies, repeated ISA-PS assessments may help identify which educational approaches are associated with the most favorable changes in attitudes toward inclusive sport. At the institutional level, aggregated ISA-PS data may inform curriculum development and provide feedback on the implementation of inclusive sport education strategies. Because inclusion is ideally embedded across the curriculum rather than addressed within a single course, repeated institutional assessments may also support the evaluation of curriculum-wide educational development and identify areas where additional educational support may be needed. Similarly, following further validation, repeated ISA-PS assessments could be used within sport organizations to evaluate inclusion-related professional development initiatives and monitor changes in practitioners' attitudes over time. In this respect, the scale may serve as a practical tool to support the alignment of educational objectives with the increasing societal and professional emphasis on inclusive sport practices, while contributing to evidence-based decisions regarding the professional development of sport professionals.

## 5 Conclusion

The present study developed and examined the Inclusive Sport Attitudes Scale for Pre-Service Sport Professionals (ISA-PS) through a multi-stage scale development and validation process. The final 16-item instrument demonstrated a theoretically interpretable four-factor structure. Confirmatory analyses further indicated that a bifactor model, comprising a general factor and four specific factors, provided the best representation of the data. The findings suggest that the ISA-PS may be suitable for assessing both overall attitudes toward inclusive sport practices and their specific aspects among pre-service sport professionals. Accordingly, the ISA-PS may complement existing measures by extending the assessment of inclusive sport attitudes beyond teacher education to pre-service sport professionals from different sport-related educational programs. It may also provide an additional tool for future research and for evaluating inclusion-related educational initiatives within sport professional education.

## Data Availability

The original contributions presented in the study are included in the article/Supplementary Material, further inquiries can be directed to the corresponding authors.

## References

[1] United Nations Department of Economic and Social Affairs. The Sustainable Development Goals Report 2023: Special Edition. (2023). Available online at: https://unstats.un.org/sdgs/report/2023/ (Accessed October 20, 2025).

[2] UNESCO. The Social Impact of Sport: Unlocking the Potential of Sport to Drive Social Transformations: Highlights. Olympic World Library. Paris: UNESCO (2024). Available online at: https://library.olympics.com/Default/doc/SYRACUSE/3416407/the-social-impact-of-sport-unlocking-the-potential-of-sport-to-drive-social-transformations-highligh (Accessed September 30, 2025).

[3] Coalter F. A Wider Social Role for Sport: Who’s Keeping the Score? London–New York: Routledge (2007).

[4] Dóczi T Gál A Sáringerné Szilárd Z. Társadalmi Befogadás a Sportban és a Sport Által (Szociális Inklúzió). Budapest: Magyar Sportmenedzsment Társaság; Magyar Sporttudományi Társaság (2014).

[5] UNESCO. International Charter of Physical Education, Physical Activity and Sport. Paris: UNESCO (2015). Available online at: https://unesdoc.unesco.org/ark:/48223/pf0000235409 (Accessed October 2, 2025).

[6] Gombás J Tóthné KK. A testkultúra és a fizikai aktivitás szerepe a fogyatékos személyek életében. In: Flamich M Hernádi I Hoffmann R Sándor A, editors. Paradigmák Örvényében. Budapest: Eötvös Loránd Tudományegyetem (2017). p. 115–24.

[7] Groff DG Lundberg NR Zabriskie RB. Influence of adapted sport on quality of life: perceptions of athletes with cerebral palsy. Disabil Rehabil. (2009) 31(4):318–26. 10.1080/09638280801976233 18608427

[8] Martin Ginis KA Van Der Ploeg HP Foster C Lai B McBride CB Ng K. Participation of people living with disabilities in physical activity: a global perspective. Lancet. (2021) 398(10298):443–55. 10.1016/S0140-6736(21)01164-8 34302764

[9] Stafylidis A. The effect of physical activity on self-esteem of people with visual impairments. J Phys Educ Sport. (2023) 23(9):2368–75. 10.7752/jpes.2023.09272

[10] Stafylidis A Chronopoulou E Papadopoulos K. The impact of independent movement and individual characteristics on self-esteem and locus of control of adults with visual impairments. Br J Vis Impair. (2026) 44(2):513–28. 10.1177/02646196251326534

[11] Barg CJ Armstrong BD Hetz SP Latimer AE. Physical disability, stigma, and physical activity in children. Int J Disabil Dev Educ. (2010) 57(4):371–82. 10.1080/1034912X.2010.524417

[12] Goodwin D Thurmeier R Gustafson P. Reactions to the metaphors of disability: the mediating effects of physical activity. Adapt Phys Activ Q. (2004) 21(4):379–98. 10.1123/apaq.21.4.379

[13] Krahé B Altwasser C. Changing negative attitudes towards persons with physical disabilities: an experimental intervention. J Community Appl Soc Psychol. (2006) 16(1):59–69. 10.1002/casp.849

[14] Lendvai L Nguyen Luu LA. A csoportközi kontaktus megélt hatása egy látó és látássérült gyermekekkel végzett vizsgálat keretében. Gyógypedagógiai Szemle. (2017) 45(4):225–40.

[15] Shapiro DR Martin JJ. Athletic identity, affect, and peer relations in youth athletes with physical disabilities. Disabil Health J. (2010) 3(2):79–85. 10.1016/j.dhjo.2009.08.004 21122772

[16] Chow WSE de Bruin K Sharma U. A scoping review of perceived support needs of teachers for implementing inclusive education. Int J Incl Educ. (2024) 28(13):3321–40. 10.1080/13603116.2023.2244956

[17] European Agency for Development in Special Needs Education. Profile of Inclusive Teachers. Odense: European Agency for Development in Special Needs Education. (2012). Available online at: https://www.european-agency.org/resources/publications/profile-inclusive-teachers (Accessed October 2, 2025).

[18] Meijer CJW Soriano V Watkins A. Inclusive education across Europe: reflections upon 10 years of work from the European agency for development in special needs education. Child Educ. (2007) 83(6):361–5. 10.1080/00094056.2007.10522951

[19] Elipe-Lorenzo P Diez-Fernández P Ruibal-Lista B López-García S. Barriers faced by people with disabilities in mainstream sports: a systematic review. Front Sports Act Living. (2025) 7:1520962. 10.3389/fspor.2025.1520962 39963671 PMC11830679

[20] King G Petrenchik T Law M Hurley P. The enjoyment of formal and informal recreation and leisure activities: a comparison of school-aged children with and without physical disabilities. Int J Disabil Dev Educ. (2009) 56(2):109–30. 10.1080/10349120902868558

[21] Silva RMF Mendonça CR Azevedo VD Memon AR Noll PRES Noll M. Barriers to high school and university students’ physical activity: a systematic review. PLoS One. (2022) 17:e0265913. 10.1371/journal.pone.0265913 35377905 PMC8979430

[22] Forlin C Loreman T Sharma U Earle C. Demographic differences in changing pre-service teachers’ attitudes, sentiments and concerns about inclusive education. Int J Incl Educ. (2009) 13(2):195–209. 10.1080/13603110701365356

[23] Kudlaček M Valkova H Sherrill C Myers B French R. An inclusion instrument based on planned behavior theory for prospective physical educators. Adapt Phys Activ Q. (2002) 19(3):280–99. 10.1123/apaq.19.3.280 28195764

[24] De Boer A Pijl SJ Minnaert A. Regular primary schoolteachers’ attitudes towards inclusive education: a review of the literature. Int J Incl Educ. (2010) 15(3):331–53. 10.1080/13603110903030089

[25] Hodge SR Tannehill D Kluge MA. Exploring the meaning of practicum experiences for PETE students. Adapt Phys Activ Q. (2003) 20(4):381–99. 10.1123/apaq.20.4.381

[26] McKay C Park JY. The impact of Paralympic Skill Lab on college student cognitive attitudes toward inclusive lifetime sport and fitness. Int J Kinesiol High Educ. (2018) 3(3):67–76. 10.1080/24711616.2018.1551732

[27] Elliott S. The effect of teachers’ attitude toward inclusion on the practice and success levels of children with and without disabilities in physical education. Int J Spec Educ. (2008) 23(3):48–55.

[28] Saloviita T. Teacher attitudes towards the inclusion of students with support needs. J Res Spec Educ Needs. (2020) 20(1):64–73. 10.1111/1471-3802.12466

[29] Farmer TW Dawes M Hamm JV Lee D Mehtaji M Hoffman AS. Classroom social dynamics management: why the invisible hand of the teacher matters for special education. Remedial Spec Educ. (2018) 39(3):177–92. 10.1177/0741932517718359

[30] Lendvai L Pap A Nguyen Luu LA. A látássérült diákok kortársakkal kapcsolatos megélt tapasztalatainak jellemzői. Gyógypedagógiai Szemle. (2019) 47(2):98–118.

[31] Filippou K Acquah EO Bengs A. Inclusive policies and practices in higher education: a systematic literature review. Rev Educ. (2025) 13(1):e70034. 10.1002/rev3.70034

[32] Anti Z Tornóczky GJ Boros S. Teaching inclusion in sports: university instructors’ insights into challenges and opportunities. Magyar Sporttudományi Szemle. (2025) 26(6):3–10.

[33] Activity Alliance. Annual Disability and Activity Survey 2023–2024. (2024). Available online at: https://www.activityalliance.org.uk/assets/000/004/907/Activity_Alliance_ADAS_Report_23-24_Accessible_original.pdf (Accessed October 20, 2025).

[34] Kielblock S Woodcock S. Who’s included and who’s not? An analysis of instruments that measure teachers’ attitudes towards inclusive education. Teach Teach Educ. (2023) 122:103922. 10.1016/j.tate.2022.103922

[35] Eagly AH Chaiken S. The Psychology of Attitudes. Fort Worth, TX: Harcourt Brace Jovanovich College Publishers (1993).

[36] Conner M Van Harreveld F Norman P. Attitude stability as a moderator of the relationships between cognitive and affective attitudes and behaviour. Br J Soc Psychol. (2022) 61(1):121–42. 10.1111/bjso.12473 34117794

[37] Braksiek M. Pre-service physical education teachers’ attitudes toward inclusive physical education: subject specificity and measurement invariance. Ger J Exerc Sport Res. (2022) 52(1):1–10. 10.1007/s12662-021-00755-1

[38] Conatser P Block M Gansneder B. Aquatic instructors’ beliefs toward inclusion: the theory of planned behavior. Adapt Phys Activ Q. (2002) 19(2):172–87. 10.1123/apaq.19.2.172 28195776

[39] Ruin S Meier S. Body and performance in (inclusive) PE settings: an examination of teacher attitudes. Int J Phys Educ. (2017) 54(3):11–23. 10.5771/2747-6073-2017-3-11

[40] Ajzen I. From intentions to actions: a theory of planned behavior. In: Kuhl J Beckmann J, editors. Action Control: From Cognition to Behavior. Berlin, Heidelberg: Springer-Verlag (1985). p. 11–39. 10.1007/978-3-642-69746-3_2

[41] Fishbein M Ajzen I. Predicting and Changing Behavior: The Reasoned Action Approach. New York: Psychology Press (2010).

[42] Antonak RF Larrivee B. Psychometric analysis and revision of the opinions relative to mainstreaming scale. Except Child. (1995) 62(2):139–49. 10.1177/001440299506200204

[43] Folsom-Meek SL Rizzo TL. Validating the Physical Educators’ Attitude Toward Teaching Individuals with Disabilities III (PEATID III) instrument for future professionals. Adapt Phys Activ Q. (2002) 19(2):141–54. 10.1123/apaq.19.2.141 28195774

[44] Forlin C Earle C Loreman T Sharma U. The sentiments, attitudes, and concerns about inclusive education revised (SACIE-R) scale for measuring pre-service teachers’ perceptions about inclusion. Except Educ Int. (2011) 21(3):50–65.

[45] Saloviita T. Measuring pre-service teachers’ attitudes towards inclusive education: psychometric properties of the TAIS scale. Teach Teach Educ. (2015) 52:66–72. 10.1016/j.tate.2015.09.003

[46] Mahat M. The development and validation of the Multidimensional Attitudes Toward Inclusive Education Scale (MATIES). Int J Spec Educ. (2008) 23(1):82–92.

[47] Ernst C Rogers MR. Development of the inclusion attitude scale for high school teachers. J Appl Sch Psychol. (2009) 25(3):305–22. 10.1080/15377900802487235

[48] Kudlaček M. Components of attitudes toward inclusion of students with physical disabilities in physical education in the revised “ATIPDPE-R” instrument/scale for prospective Czech educators. Acta Univ Palacki Olomuc Gymn. (2007) 37(1):13–8.

[49] Hodge SR Murata NM Kozub FM. Physical educators’ judgments about inclusion: a new instrument for pre-service teachers. Adapt Phys Activ Q. (2002) 19(4):435–52. 10.1123/apaq.19.4.435 28195787

[50] Yuker HE Block JR Campbell WJ. A Scale to Measure Attitudes Toward Disabled Persons. Albertson, NY: Human Resources Foundation (1960). 10.1037/t16118-000

[51] Findler L Vilchinsky N Werner S. The Multidimensional Attitudes Scale Toward Persons with Disabilities (MAS): construction and validation. Rehabil Couns Bull. (2007) 50(3):166–76. 10.1177/00343552070500030401

[52] Li Q Wang L Qi J Li C. Pre-service physical education teacher concerns about teaching students with disabilities: instrument modification and validation. Int J Disabil Dev Educ. (2022) 69(3):791–806. 10.1080/1034912X.2020.1719986

[53] Kozub FM Porretta DL. Interscholastic coaches’ attitudes toward integration of adolescents with disabilities. Adapt Phys Activ Q. (1998) 15(4):328–44. 10.1123/apaq.15.4.328

[54] Ajzen I. Constructing a Theory of Planned Behavior Questionnaire. Amherst, MA: University of Massachusetts (2006). Available online at: https://people.umass.edu/aizen/pdf/tpb.measurement.pdf (Accessed October 6, 2024).

[55] Chyung SY Roberts K Swanson I Hankinson A. Evidence-based survey design: the use of a midpoint on the Likert scale. Perform Improv. (2017) 56(10):15–23. 10.1002/pfi.21727

[56] Garland R. The mid-point on a rating scale: is it desirable? Mark Bull. (1991) 2:66–70.

[57] Beaton DE Bombardier C Guillemin F Ferraz MB. Guidelines for the process of cross-cultural adaptation of self-report measures. Spine. (2000) 25(24):3186–91. 10.1097/00007632-200012150-00014 11124735

[58] Bogardus ES. Measuring social distances. J Appl Sociol. (1925) 9:299–308.

[59] Faragó L Kende A. Az elnyomás támogatása vagy az esélyegyenlőség ellenzése? Az új Szociális Dominancia Orientáció Skála (SDO7) vizsgálata. Alkalmazott Pszichológia. (2017) 17(1):115–35. 10.17627/ALKPSZICH.2017.1.115

[60] Ho AK Sidanius J Kteily N Sheehy-Skeffington J Pratto F Henkel KE. The nature of social dominance orientation: theorizing and measuring preferences for intergroup inequality using the new SDO7 scale. J Pers Soc Psychol. (2015) 109(6):1003–28. 10.1037/pspi0000033 26479362

[61] Kim HY. Statistical notes for clinical researchers: assessing normal distribution (2) using skewness and kurtosis. Restor Dent Endod. (2013) 38(1):52–4. 10.5395/rde.2013.38.1.52 23495371 PMC3591587

[62] Costello AB Osborne JW. Best practices in exploratory factor analysis: four recommendations for getting the most from your analysis. Pract Assess Res Eval. (2005) 10(7):1–9. 10.7275/jyj1-4868

[63] Bartlett MS. Tests of significance in factor analysis. Br J Stat Psychol. (1950) 3(2):77–85. 10.1111/j.2044-8317.1950.tb00285.x

[64] Kaiser HF. An index of factorial simplicity. Psychometrika. (1974) 39(1):31–6. 10.1007/BF02291575

[65] DeVellis RF. Scale Development: Theory and Applications. 4th ed Thousand Oaks, CA: Sage (2017).

[66] Tabachnick BG Fidell LS. Using Multivariate Statistics. 7th ed Harlow, UK: Pearson (2019).

[67] Nunnally JC Bernstein IH. Psychometric Theory. 3rd ed New York: McGraw-Hill (1994).

[68] Li CH. Confirmatory factor analysis with ordinal data: comparing robust maximum likelihood and diagonally weighted least squares. Behav Res Methods. (2016) 48(3):936–49. 10.3758/s13428-015-0619-7 26174714

[69] Kline RB. Principles and Practice of Structural Equation Modeling. 4th ed New York: Guilford Press (2016).

[70] Hu L Bentler PM. Cutoff criteria for fit indexes in covariance structure analysis: conventional criteria versus new alternatives. Struct Equ Modeling. (1999) 6(1):1–55. 10.1080/10705519909540118

[71] Brown TA. Confirmatory Factor Analysis for Applied Research. 2nd ed New York: Guilford Press (2015).

[72] Xia Y Yang Y. RMSEA, CFI, and TLI in structural equation modeling with ordered categorical data: the story they tell depends on the estimation methods. Behav Res. (2019) 51:409–28. 10.3758/s13428-018-1055-2 29869222

[73] Reise SP. The rediscovery of bifactor measurement models. Multivariate Behav Res. (2012) 47(5):667–96. 10.1080/00273171.2012.715555 24049214 PMC3773879

[74] Rodriguez A Reise SP Haviland MG. Evaluating bifactor models: calculating and interpreting statistical indices. Psychol Methods. (2016) 21(2):137–50. 10.1037/met0000045 26523435

[75] Hair JF Jr Black WC Babin BJ Anderson RE. Multivariate Data Analysis. 8th ed. Boston, MA: Cengage Learning. (2019).

[76] Chen FF West SG Sousa KH. A comparison of bifactor and second-order models of quality of life. Multivariate Behav Res. (2006) 41(2):189–225. 10.1207/s15327906mbr4102_5 26782910

[77] Fornell C Larcker DF. Evaluating structural equation models with unobservable variables and measurement error. J Mark Res. (1981) 18(1):39–50. 10.2307/3151312

[78] Clark LA Watson D. Constructing validity: new developments in creating objective measuring instruments. Psychol Assess. (2019) 31(12):1412–27. 10.1037/pas0000626 30896212 PMC6754793

[79] Cohen J. Statistical Power Analysis for the Behavioral Sciences. 2nd ed Hillsdale, NJ: Lawrence Erlbaum Associates (1988).

[80] Siegel S Castellan NJ Jr. Nonparametric Statistics for the Behavioral Sciences. 2nd ed New York: McGraw-Hill (1988).

[81] Meier S Ruin S. Creation and validation of the pre-service PE teachers’ attitudes towards inclusive physical education scale (ATIPE). Int J Phys Educ. (2019) 56(1):21–32. 10.5771/2747-6073-2019-1-21

[82] Hair JF Black WC Babin BJ Anderson RE. Multivariate Data Analysis. 7th ed Upper Saddle River, NJ: Pearson (2010).

[83] Wrightsman LS. Interpersonal trust and attitudes toward human nature. In: Robinson JP Shaver PR Wrightsman LS, editors. Measures of Personality and Social Psychological Attitudes. San Diego, CA: Academic Press (1991). p. 373–412.

[84] Kárász JT Nagybányai Nagy O Széll K Takács S. Cronbach-alfa: Vele vagy nélküle? Magyar Pszichológiai Szemle. (2022) 77(1):81–98. 10.1556/0016.2022.00004

[85] Navarro-Mateu D Franco-Ochoa J Valero-Moreno S Prado-Gascó V. To be or not to be an inclusive teacher: are empathy and social dominance relevant factors to positive attitudes towards inclusive education? PLoS One. (2019) 14(12):e0225993. 10.1371/journal.pone.0225993 31821354 PMC6903715

[86] Armitage CJ Conner M. Efficacy of the theory of planned behaviour: a meta-analytic review. Br J Soc Psychol. (2001) 40(4):471–99. 10.1348/014466601164939 11795063

[87] Gilor O Katz M. Pre-service teachers’ willingness to engage in inclusive teaching: an explanatory model. J Int Spec Needs Educ. (2018) 22(2):77–89. 10.9782/17-00011

[88] Fiske ST Cuddy AJC Glick P Xu J. A model of (often mixed) stereotype content: competence and warmth respectively follow from perceived status and competition. J Pers Soc Psychol. (2002) 82(6):878–902. 10.1037/0022-3514.82.6.878 12051578

[89] Indriani R Sulastri A Husni M Hadi YA. The influence of inclusive education on students’ social skills and self-esteem at SDN 1 Gereneng in the academic year 2025/2026. Modeling J Program Stud PGMI. (2025) 12(3):294–308. 10.69896/modeling.v12i3.2970

[90] O'Donovan MA Wedgwood N Westermann G Rillotta F Aitken T. Personal, social and vocational outcomes of inclusive university for people with intellectual disability. J Intellect Disabil Res. (2025) 69:1448–73. 10.1111/jir.70042 41016861 PMC12580482

[91] McCracken T Chapman S Piggott B. Inclusion illusion: a mixed-methods study of preservice teachers and their preparedness for inclusive schooling in Health and Physical Education. Int J Incl Educ. (2023) 27(4):507–25. 10.1080/13603116.2020.1853259

[92] Taliaferro AR Hammond L Bowen K. Preservice physical educators’ self-efficacy beliefs toward inclusion: the impact of coursework and practicum. Adapt Phys Activ Q. (2015) 32(1):49–67. 10.1123/apaq.2013-0112 25544720

[93] Di Nardo M Kudláček M Tafuri D Sklenářiková J. Attitudes of preservice physical educators toward individuals with disabilities at University Parthenope of Napoli. Acta Gymn. (2014) 44(4):211–21. 10.5507/ag.2014.022

[94] Hodge SR Davis R Woodard R Sherrill C. Comparison of practicum types in changing preservice teachers’ attitudes and perceived competence. Adapt Phys Activ Q. (2002) 19(2):155–71. 10.1123/apaq.19.2.155 28195772

[95] Sharma U Shaukat S Furlonger B. Attitudes and self-efficacy of pre-service teachers towards inclusion in Pakistan. J Res Spec Educ Needs. (2015) 15(2):97–105. 10.1111/1471-3802.12071

[96] Parasuram K. Variables that affect teachers’ attitudes towards disability and inclusive education in Mumbai, India. Disabil Soc. (2006) 21(3):231–42. 10.1080/09687590600617352

[97] Romero-Contreras S Garcia-Cedillo I Forlin C Lomelí-Hernández KA. Preparing teachers for inclusion in Mexico: how effective is this process? J Educ Teach. (2013) 39(5):509–22. 10.1080/02607476.2013.836340

[98] Liu Y Kudláček M Ješina O. The influence of Paralympic School Day on children’s attitudes towards people with disabilities. Acta Gymn. (2010) 40(2):63–9.

[99] Reina R López V Jiménez M García-Calvo T Hutzler Y. Effects of awareness intervention on children’s attitudes toward peers with a visual impairment. Int J Rehabil Res. (2011) 34(3):243–8. 10.1097/MRR.0b013e3283487f49 21694601

[100] Zajonc RB. Attitudinal effects of mere exposure. J Pers Soc Psychol Monogr Suppl. (1968) 9(2 Pt 2):1–27. 10.1037/h0025848

[101] Allport GW. The Nature of Prejudice. Reading, MA: Addison-Wesley (1954).

