# Supplementary material for: Development and validation of the inclusive sport attitudes scale for pre-service sport professionals (ISA-PS)
Source: Front Sports Act Living. 2026 Jul 29;8:1917186. doi: 10.3389/fspor.2026.1917186 (PMC13461791; doi:10.3389/fspor.2026.1917186)
Supplement: Supplementary file 1 [file Supplementaryfile1.docx]

Supplementary Material

# 1 ****Supplementary File S1. ISA-PS Manual and Item Sets (Hungarian Version and English Translation)****

This supplementary file contains the ISA-PS manual, including participant instructions, key definitions, response options, scoring information, and the complete item sets in Hungarian and English. The Hungarian version was psychometrically validated in the present study. The English version is provided as a translation developed through a cross-cultural adaptation procedure and has not yet undergone psychometric validation.

**Az Inkluzív Sportoltatás Attitűdskála Leendő Sportszakemberek verziója (ISA-LS)**

**Instrukciók** (a skála csak a következő fogalmi bevezetővel együtt alkalmazható):

**Az alábbiakban az inklúzió definícióját, majd egy rövid ismertetőt fog olvasni.**

**Kérjük, ezeket a szempontokat figyelembe véve válaszoljon a következő kérdésekre!**

*Az inklúzió a sokféleséget természetesként kezelő, minden résztvevő számára egyenlő hozzáférést, befogadást biztosító szemléletmód, melynek központjában a sokféleség értékként való kezelése áll, így képes meggátolni a társadalmi kirekesztést, kirekesztődést.*

A kövezőkben inkluzív sportoltatással, inkluzív sportfoglalkozásokkal kapcsolatos állításokat olvashat. Jelen felmérésben inkluzív sportfoglalkozás alatt olyan szervezett sporttevékenységet értünk, amely az inkluzió szemléletének megfelelően lehetőséget biztosít fogyatékos és nem fogyatékos emberek együttes részvételére, szem előtt tartva a különféle képességekkel rendelkező résztvevők igényeit.

**Válaszadáskor *sportoltatás* alatt arra a tevékenységre gondoljon, amelyet sportszakemberként a képzésének megfelelően végezni fog. Pl. testnevelésóra megtartása, edzés, táboroztatás, sportesemény szervezése stb.**

(Szerzői megjegyzés: az instrukciók utolsó, kiemelt bekezdése a vizsgálat célcsoportjához igazítható, a *sportoltatás* értelmezése a kitöltők körének megfelelően módosítható.)

**Kérjük, értékelje 1-től 6-ig tartó skálán, hogy mennyire ért egyet az alábbi állításokkal!** (1=egyáltalán nem értek egyet; 2=többnyire nem értek egyet; 3=inkább nem értek egyet; 4=inkább egyetértek; 5=többnyire egyetértek; 6= teljes mértékben egyetértek)

|  | 1 | 2 | 3 | 4 | 5 | 6 |
| --- | --- | --- | --- | --- | --- | --- |
| 1. A fogyatékos emberek is képesek jól teljesíteni egy inkluzív sportfoglalkozáson. |  |  |  |  |  |  |
| 2. A speciális szükségletek miatt a fogyatékos embereknek az a legjobb, ha külön sportfoglalkozáson vesznek részt. |  |  |  |  |  |  |
| 3. A sportfoglalkozásokon rendszerint túl sok a résztvevő és túl kevés a sportszakember ahhoz, hogy az inkluzivitás megvalósulhasson. |  |  |  |  |  |  |
| 4. Egy inkluzív sportfoglalkozás gyakorlataiból és módszereiből a fogyatékos és nem fogyatékos résztvevők egyaránt profitálhatnak. |  |  |  |  |  |  |
| 5. Örömmel tölt el, hogy egy inkluzív sportfoglalkozás minden résztvevő számára képes megtanítani az együttműködést, valamint a hasonlóságok és különbségek elfogadását. |  |  |  |  |  |  |
| 6. A fogyatékos emberek sportoltatásával csak speciális képzettséggel rendelkező szakemberek tudnak hatékonyan foglalkozni. |  |  |  |  |  |  |
| 7. Úgy érzem, kevesebbet tudnék nyújtani a résztvevőknek, csökkenne a szakmai teljesítményem, ha fogyatékos emberek is részt vennének a foglalkozásomon. |  |  |  |  |  |  |
| 8. Örömmel részt vennék további képzéseken, hogy bővítsem a fogyatékos emberek sportoltatásával kapcsolatos tudásomat. |  |  |  |  |  |  |
| 9. Szívesen elsajátítom és beszerzem a fogyatékos emberek igényeihez igazodó módszereket és eszközöket. |  |  |  |  |  |  |
| 10. Nagyfokú stresszt okozna számomra, ha fogyatékos emberek is részt vennének a sportfoglalkozásomon. |  |  |  |  |  |  |
| 11. Képtelen leszek fenntartani a sportfoglalkozás színvonalát, ha azon fogyatékos emberek is részt vesznek. |  |  |  |  |  |  |
| 12. Szeretnék inkluzív sportfoglalkozásokat tartani, mert a különféle képességekkel rendelkező résztvevők bevonásának köszönhetően jobb sportszakember válik belőlem. |  |  |  |  |  |  |
| 13. Képesnek érzem magam arra, hogy fogyatékos emberek részvételével tartsak inkluzív sportfoglalkozást. |  |  |  |  |  |  |
| 14. Félek, hogy a munkám során nem kapnék elég ösztönzést (például extra juttatásokat, megbecsülést), ha fogyatékos emberekkel foglalkoznék. |  |  |  |  |  |  |
| 15. Nem vállalom azt a többletmunkát, ami a fogyatékos emberek részvételével tartott sportfoglalkozások tervezésével és előkészítésével jár. |  |  |  |  |  |  |
| 16. Szívesen fogadnám a fogyatékos résztvevőket a sportfoglalkozásomon. |  |  |  |  |  |  |
| **Inkluzív sportoltatásra vonatkozó szándék** | | | | | | |
| 17. Szándékomban áll fogyatékos és nem fogyatékos emberek együttes részvételével inkluzív sportfoglalkozásokat tartani. |  |  |  |  |  |  |

**Fordított tételek**: 2., 3., 6., 7., 10., 11., 14., 15.

**Skálaképzési útmutató**:

**Pozitív hajlandóság**: 8., 9., 12., 13., 16.

**Negatív komponensek a sportszakemberek számára**: 7., 10., 11., 14., 15.

**Pozitív komponensek a résztvevők számára**: 1., 4., 5.

**Inklúzió korlátai**: 2., 3., 6.

A skálaképzés az egyes alskálákhoz (faktorok) tartozó tételekre adott válaszok pontszámainak összeadásával történik. A magasabb pontszám kedvezőbb inkluzív sportoltatással kapcsolatos attitűdöt jelez.

A 17. kérdést, ami nem a skála része, kérjük az ISA-LS skála végén beilleszteni az inkluzív sportoltatásra vonatkozó szándék felmérésére.

**Inclusive Sport Attitudes Scale – for Pre-Service Sport Professionals (ISA-PS)**

**Manual** *(the scale may only be administered together with the following conceptual introduction):*

INSTRUCTIONS:

**In the description below you will read the definition of inclusion followed by a brief introduction.**

**Please think back to these considerations when answering the following questions.**

***Inclusion is an approach*** *that treats diversity as natural and guarantees equity and a sense of belonging for all participants. At its core is the recognition of diversity as a value, which enables it to prevent social exclusion and marginalisation.*

In the following section, you will read statements related to **inclusive sports sessions**.

In this survey, **inclusive sports sessions** refer to organized sport programs that, in accordance with the principles of inclusion, enable people with and without disabilities to participate together, while considering the needs of participants with diverse abilities.

When responding, please consider ‘**sports sessions’** as the type of activities you will carry out as a sports professional in line with your training. For example, conducting PE classes, training sessions, camps, or organising sports events.

**On a scale of 1-6, please rate how much do you agree with the following statements!** (1 = Strongly disagree; 2 = Mostly disagree; 3 = Somewhat disagree; 4 = Somewhat agree; 5 = Mostly agree; 6 = Strongly agree)

|  | 1 | 2 | 3 | 4 | 5 | 6 |
| --- | --- | --- | --- | --- | --- | --- |
| 1. People with disabilities are also capable of performing well in inclusive sports sessions. |  |  |  |  |  |  |
| 2. Due to their special needs, it is best for people with disabilities to participate in separate sports sessions. |  |  |  |  |  |  |
| 3. In sports sessions, there are usually too many participants and too few sports professionals to ensure inclusion. |  |  |  |  |  |  |
| 4. Both participants with and without disabilities can benefit from the practices and methods used in inclusive sports sessions. |  |  |  |  |  |  |
| 5. I am pleased that an inclusive sports session can teach all participants cooperation and embracing similarities and differences. |  |  |  |  |  |  |
| 6. Only professionals with special training are able to effectively conduct sports activities for people with disabilities. |  |  |  |  |  |  |
| 7. I feel that I could offer less to the participants and that my professional performance would decrease if people with disabilities also participated in my sports sessions. |  |  |  |  |  |  |
| 8. I would gladly attend further training to expand my knowledge of conducting sports activities for people with disabilities. |  |  |  |  |  |  |
| 9. I would be pleased to learn and acquire the practices and tools adapted to the needs of people with disabilities. |  |  |  |  |  |  |
| 10. It would cause me significant stress if people with disabilities participated in my sports sessions. |  |  |  |  |  |  |
| 11. I will be unable to maintain the quality of my sports sessions if people with disabilities also participate in them. |  |  |  |  |  |  |
| 12. I would like to conduct inclusive sports sessions because including participants with diverse abilities would help me become a better sports professional. |  |  |  |  |  |  |
| 13. I feel capable of conducting inclusive sports sessions with the participation of people with disabilities. |  |  |  |  |  |  |
| 14. I am afraid that I would not receive enough incentives (additional allowance or appreciation) if I worked with people with disabilities. |  |  |  |  |  |  |
| 15. I am not willing to take on the extra work involved in planning and preparing sports sessions that include people with disabilities. |  |  |  |  |  |  |
| 16. I would be happy to welcome people with disabilities to my sports sessions. |  |  |  |  |  |  |
| **Intention toward Inclusive Sport (IIS)** | | | | | | |
| 17. I intend to conduct inclusive sport sessions with the participation of people with and without disabilities. |  |  |  |  |  |  |

**Reverse-scored items:** 2, 3, 6, 7, 10, 11, 14, 15.

**Scoring instructions:**

- **Positive Willingness:** Items 8, 9, 12, 13, 16.
- **Negative Components for Sport Professionals:** Items 7, 10, 11, 14, 15.
- **Positive Components for Participants:** Items 1, 4, 5.
- **Barriers to Inclusion:** Items 2, 3, 6.

Scale scores are calculated by summing the item scores belonging to each subscale (factor). Higher scores indicate a more favorable attitude toward inclusive sport.

Item 17, which is not part of the scale, should be placed at the end of the ISA-PS scale to assess intention toward inclusive sport.
